# H3K27me3 modulates trained immunity of monocytes in HDM-allergic diseases

**DOI:** 10.3389/fimmu.2025.1572796

**Published:** 2025-05-28

**Authors:** Lingli Han, Lin Li, Liangjiao Yao, Huaqin Bu, Yajie Tian, Qifan Li, Ke Zhu, Haili Yao, Xiaochuan Wang, Maoxiang Qian, Wei Lu, Jinqiao Sun

**Affiliations:** ^1^ Department of Clinical Immunology, Children's Hospital of Fudan University, National Children's Medical Center, Shanghai, China; ^2^ National Health Commission (NHC) Key Laboratory of Neonatal Diseases, National Children's Medical Center, Shanghai, China; ^3^ Institute of Pediatrics and Department of Hematology and Oncology, Children’s Hospital of Fudan University, National Children’s Medical Center, and Shanghai Key Laboratory of Medical Epigenetics, International Co-Laboratory of Medical Epigenetics and Metabolism (Ministry of Science and Technology), Institutes of Biomedical Sciences, Fudan University, Shanghai, China; ^4^ Shanghai Institute of Nutrition and Health, University of Chinese Academy of Sciences, Chinese Academy of Sciences, Shanghai, China

**Keywords:** HDM, monocytes, inflammation, KDM6B, H3K27me3

## Abstract

**Background:**

Monocytes have been confirmed to increase in persistently food-allergic children. A phenomenon of innate immune memory, called trained immunity, has also been observed in monocytes from allergic children. However, the underlying mechanism remains poorly understood.

**Methods:**

We enrolled a cohort of HDM-allergic children alongside age-matched healthy controls and established an HDM-sensitized allergic mouse model. Flow cytometric analyses were conducted to quantify monocyte frequencies in clinical cohorts and experimental animals. We performed integrated transcriptomic profiling via RNA-seq combined with chromatin occupancy analysis using CUT&Tag technology in parallel human and murine samples to elucidate the molecular mechanisms.

**Results:**

In our study, we demonstrated a reduced H3K27me3 methylation level accompanied by an increased proportion and a proinflammatory transcriptional memory in monocytes from house dust mite (HDM)-allergic human subjects. The same transcriptional and epigenetic phenotype was also confirmed in HDM-sensitized mice. Finally, the administration of GSK-J4, which upregulates H3K27me3 level in murine monocytes, attenuated the inflammatory response *in vitro* and *in vivo*.

**Conclusions:**

Our study confirms that H3K27me3 methylation modulates the trained immunity in monocytes and regulates HDM-allergic diseases through an inflammatory-dependent mechanism.

## Introduction

The prevalence of airway allergic diseases, including allergic rhinitis and asthma, is rising rapidly worldwide (1). It’s estimated that almost 40% of children were affected by allergic rhinitis, and 4.3% of the population was affected by asthma worldwide (2, 3). House dust mite (HDM) has been confirmed as the most common allergen triggering allergic rhinitis and asthma (4). *Dermatophagoides farina* (Der f*)* and *Dermatophagoides pteronyssinus* (Der p) HDM are the two potent components that threaten human health, affecting more than half a billion people worldwide (5, 6).

The pandemic of allergic diseases is propelling research into underlying mechanisms. Although CD4^+^ Th2 cell polarization and crosslinking of IgE antibodies in basophils and mast cells have been shown to dominate the pathogenesis of allergic diseases for decades (7), innate immune cells such as monocytes have been reported to increase in food-allergic children (8, 9). However, the role of this increased monocyte population in allergic diseases has not been illuminated.

Recent studies have demonstrated that innate immune cells possess a form of memory state termed trained immunity. Trained immunity elevates the response potential of innate immune cells during the second challenge, following exogenous and endogenous stimulation (10). The existence of trained immunity has been identified in the *Bacillus Calmette–Guerin* vaccination and other diseases (11–14). In allergic diseases, the phenomenon of trained immunity has been observed in monocytes of food-allergic children. The frequency of monocytes was increased in children with persistent food allergy, but not in those with transient food allergy. Besides, the monocytes in persistent food-allergic children showed exacerbated inflammatory cytokine secretion after low-dose and long-term LPS stimulation (9). The role of monocytes has also been investigated in HDM-sensitized murine models. IL-4Rα^+^ monocytes and macrophages drive a self-sustaining type 2 inflammatory loop in HDM-sensitized mice (15). Monocyte-derived dendritic cells (moDCs) are pivotal in initiating Th2 immunity against HDM allergens through antigen presentation and co-stimulatory signaling (16). Additionally, monocyte-derived macrophages exhibit TNF-dependent memory responses in both HDM-sensitized mice and allergic patients, perpetuating inflammatory cascades (17). Nevertheless, the detailed mechanism of trained immunity modulation in monocytes that drives the development of persistent allergic diseases has not been elucidated.

Given that epigenetic modification is the molecular basis of trained immunity (18–20), in our current study, we recruited recurrent HDM-allergic patients and evaluated their cell frequencies, transcriptional profiles, and epigenetic alterations of monocytes compared to healthy controls. We identified that Histone 3 demethylase, KDM6B, was a critical epigenetic factor regulating the development of trained immunity. In allergic monocytes from patients and animal models, KDM6B reduces the level of H3K27me3, a suppressive histone marker, to inhibit proinflammatory gene expression.

## Material and methods

### Subjects

Subjects allergic to HDM and healthy allergy-free controls were recruited from March 2022 to November 2022 at Children’s Hospital of Fudan University. The patients were clinically diagnosed with HDM (Der p and/or Der f) allergic rhinitis and asthma based on confirmed exposure history, typical clinical symptoms, total IgE levels, and the presence of allergen-specific IgE as measured with Immunocap (Pharmacia, Uppsala, Sweden). Patients and healthy controls were excluded if they had any other acute or chronic diseases. Additional exclusion criteria included immunodeficiency and drug allergies. Age-matched healthy controls were verified to be free of allergic history and symptoms through a detailed questionnaire.

### Mouse models of lung inflammation

Age-matched female C57BL/6 wild-type mice, aged 6 to 8 weeks, were bred in a pathogen-free facility. The mice were intratracheally sensitized with 50 μg Der p-HDM, which underwent endotoxin removal (Greerlabs, USA), for 3 days. Following sensitization, the mice were allowed to rest for 11 days and challenged with 20 μg HDM over 4 consecutive days (Der p-A). The blank control group was consistently challenged with an equivalent phosphate buffer solution (PBS). The mice were sacrificed within 24 hours of the last challenge. The *in vivo* administration of GSK-J4 adhered to the following protocol: wild-type mice were initially sensitized with 50 μg Der p-HDM. Subsequently, the mice received daily intraperitoneal injections of GSK-J4 (20 mg/kg body weight, diluted with 5% DMSO, 40% PEG-300, 5% Tween-80, and 50% double distilled water) for 14 consecutive days. During the final 4 days of the treatment period, the mice were also subjected to airway rechallenge with 20 μg of HDM.

### Ethic and regulatory approval of human and animal subjects

The study involving human subjects received approval from the Ethics Committee of the Children’s Hospital of Fudan University. Blood samples from patients and healthy controls were collected according to approved protocols (Ethics Approval Number (2022): No.27). Mouse experimental protocols were approved by the Institutional Biomedical Research Ethics Committee of the Shanghai Institute of Nutrition and Health Science, Chinese Academy of Sciences.

### Histology

The lungs were fixed in 4% paraformaldehyde for 24 hours, followed by dehydration, waxing, and embedding in paraffin. The embedded tissues were then cut into sections and stained with hematoxylin-eosin (H&E) and periodic acid-Schiff (PAS).

### ELISA

Serum levels of IL-4, IL-5, and total IgE were determined in murine specimens (n=6–8 per group) using the ELISA MAX™ Deluxe Set (BioLegend, USA) according to the manufacturer’s protocol. Briefly, 96-well plates were coated with capture antibodies and maintained at 4°C overnight. After blocking non-specific binding sites, serum samples (1:3 dilution) and standard calibrators were incubated simultaneously in separate wells for 2 hours at room temperature. Following four wash cycles with PBS-T buffer, plates were sequentially treated with biotinylated detection antibodies and avidin-horseradish peroxidase (HRP) conjugate. Colorimetric development was initiated by adding tetramethylbenzidine (TMB) substrate and was terminated when optimal chromogenic intensity was achieved. Optical density at 450 nm was measured using a microplate reader, with standard curve generation and data analysis performed through linear regression modeling in GraphPad Prism software (version 9.0).

### Flow cytometric analysis

Peripheral blood samples from 22 HDM-allergic pediatric patients and 18 healthy controls were processed as follows: A total of 100 μL of fresh whole blood was stained with fluorochrome-conjugated antibodies against CD45 (HI30), CD14 (M5E2), CD16 (3G8), and HLA-DR (L243) to identify classical, intermediate, and non-classical monocyte subsets. After erythrocyte lysis, cells were resuspended in PBS and analyzed using a CytoFLEX LX Flow Cytometer (Beckman Coulter, USA).

Bronchoalveolar lavage fluid (BALF) from HDM-allergic mice was incubated with anti-CD45 (30-F11), anti-Siglec-F (S17007L), anti-CD11c (N418), anti-CD11b (30-F11), anti-Ly6C (HK1.4), and anti-F4/80 (BM8) to label eosinophils, monocytes, and macrophages. Bone marrow erythrocytes were lysed. All flow cytometry data were acquired using standardized instrument settings and analyzed with FlowJo software (v10.0). Cell population frequencies of monocytes in human subjects and murine models were quantified through hierarchical gating strategies (detailed in **Supplementary Figure S2**) and statistically analyzed using GraphPad Prism (version 9.0).

### Monocyte isolation

Human peripheral blood mononuclear cells (PBMCs) were enriched using Ficoll-Paque. Monocytes were isolated with CD14 MicroBeads (Miltenyi, Germany). Mouse bone marrow monocytes were enriched using the EasySep Mouse Monocyte Isolation Kit (STEMCELL Technologies, Canada).

### *In vitro* stimulation and culture

Monocytes isolated from HDM-allergic pediatric patients (n=17) and healthy controls (n=11) were stimulated with 10 ng/mL LPS for 4 hours, followed by TRIzol-based cell lysis and RNA extraction for subsequent transcriptome profiling.

Monocytes were magnetically isolated from wild-type C57BL/6J mouse bone marrow using the EasySep™ Mouse Monocyte Isolation Kit (STEMCELL Technologies, Canada) according to the manufacturer’s protocol. Purified cells were primed with 10 μg/mL HDM extract (Greer Laboratories, USA) in complete RPMI-1640 medium for 16 h at 37°C/5% CO_2_. After three consecutive PBS washes, cells were incubated with 5 μM GSK-J4 (Selleck, USA), a selective dual inhibitor targeting both KDM6A and KDM6B histone demethylases, for a 24-hour period under standard culture conditions (37°C, 5% CO_2_) (21). Subsequent secondary stimulation involved replenishing the culture system with 10 μg/mL HDM for an additional 24 h. Terminally treated cells were harvested using TRIzol™ Reagent for downstream RNA analysis.

### Quantitative PCR

Total RNA was extracted using TRIzol reagent (Thermo Fisher Scientific). cDNA was synthesized with Transcript RT Master Mix (Takara). Quantitative PCR was performed using Takara SYBR Fast qPCR Mix (Takara). β-actin served as an internal reference. Fold changes were calculated via the 2^-ΔΔCT method. The primer sequences are as follows:

*Tnf*: Forward: 5’- CCCTCACACTCAGATCATCTTCT-3’,

Reverse: 5’-GCTACGACGTGGGCTACAG-3’;

*Il1b*: Forward: 5’-GCAACTGTTCCTGAACTCAACT-3’,

Reverse: 5’-ATCTTTTGGGGTCCGTCAACT-3’.

*Il6:* Forward: 5’- CCAAGAGGTGAGTGCTTCCC-3’,

Reverse: 5’-CTGTTGTTCAGACTCTCTCCCT-3’;

*Il10:* Forward: 5’-GCTCTTACTGACTGGCATGAG-3’,

Reverse: 5’-CGCAGCTCTAGGAGCATGTG-3’;

### Library construction and sequencing

Total RNA was isolated using the RNAmini kit (Qiagen, Germany). RNA libraries were constructed using the TruSeq RNA sample preparation kit (Illumina, USA). Paired-end Cleavage Under Targets and Tagmentation (CUT&Tag) libraries were generated using the Hyperactive Universal CUT&Tag Assay Kit for Illumina (Vazyme, China) and sequenced on the Illumina Novoseq 6000 platform.

### Bioinformatic analysis

#### RNA-seq data process

RNA-seq reads, after removing adapters and low-quality reads using Trim-galore (0.6.7, https://github.com/FelixKrueger/TrimGalore), were aligned to the reference human genome GRCh37 and mouse genome mm10 with STAR software (2.7.9a). Transcripts were assembled and quantified using Stringtie software (1.3.5) (20, 21). Differential expression analysis was conducted on protein-coding genes utilizing DESeq2 (1.32.0). Genes with an FDR value less than 0.05 were selected. GO and KEGG enrichment analyses were performed with clusterProfiler (4.0.5) (22). Heatmaps of gene expression after rlog normalization across different samples were plotted using ggplot2 (3.3.5) (23).

#### CUT&Tag analysis

The CUT&Tag data was aligned to the reference human genome GRCh37 and mouse genome mm10 using Bowtie2 tools. After removing unmapped and duplicate reads, the MACS2 (2.2.7.1) software was used to call peaks to identify H3K27me3 binding regions. The common binding regions were identified using the multiinter tool of BEDTools (2.30.0), and the CPM value for each common region was calculated. Regions showing at least a two-fold increase or decrease compared to the reference group were selected as differential binding regions, and functional enrichment analysis of these regions was performed using the online tool GREAT (24) (http://great.stanford.edu/public/html/). The bamCoverage tool of deepTools (3.5.1) was used to convert the aligned BAM files to bigWig format after removing duplicates, and the computeMatrix tool was employed to calculate signals in the regions of protein-coding genes (25).

#### Statistical analysis

Statistical analyses were conducted using unpaired two-tailed Student’s t-tests for intergroup comparisons in monocyte flow cytometry, serum ELISA quantification of IL-4/IL-5/IgE, and qPCR-based gene expression analysis (n=6–8 mice per group; n=22 patients and 18 healthy controls). For multiple group comparisons in murine BALF subset frequency analysis, we applied Holm-Sidak-adjusted t-tests to control for family-wise error rates (n=6–8 per group). All computations were executed in GraphPad Prism 9.4.1, with the standard error of the mean (SEM) reported across all assays. A significance threshold of P<0.05 was applied throughout the study.

## Results

### Monocytes showed persistent transcriptional proinflammatory memory in HDM-allergic children

**Figure 1:**
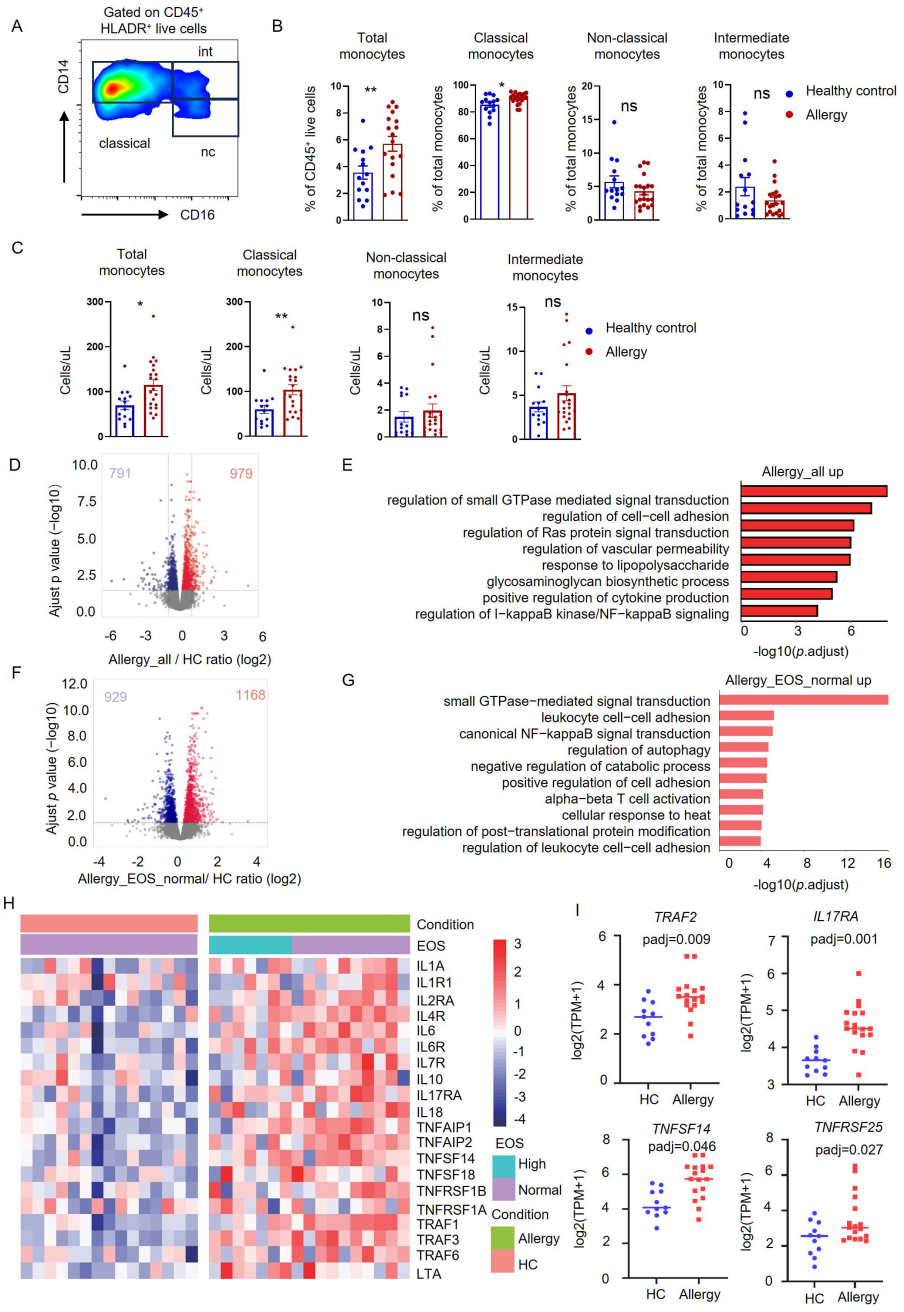
Monocytes showed transcriptional proinflammatory memory in HDM-allergic children. **(A)** Representative FACS plots of peripheral monocyte subsets (CD14^high^ CD16^-^ classical monocytes, CD14^high^ CD16^+^ intermediate monocytes and CD14^low^ CD16^+^ non-classical monocytes), defined by CD14 and CD16 surface markers. **(B)** Statistical comparison of cell frequency of total monocytes and monocyte subsets in peripheral blood of HDM-allergic children (n=22) and healthy controls (n=18). *p<0.05; **p<0.01 and ns no significance. p values were determined using unpaired two-tailed Student’s t-tests. **(C)** Statistical comparison of cell counts of total monocytes and monocyte subsets in peripheral blood of HDM-allergic children and healthy controls. *p<0.05; **p<0.01 and *ns* no significance. p values were determined using unpaired two-tailed Student’s t-tests. **(D)** Volcano plots showing the 979 upregulated genes and 791 downregulated genes in monocytes of all HDM-allergic children (n=18) compared to healthy controls (HC, n=14) (p-adj<0.05 and FDR<0.05). **(E)** Top overrepresented GO enrichment analysis and KEGG pathways showing upregulated (red) in classical monocytes from all allergic-children(n=18) and healthy control (n=14). **(F)** Volcano plots showing the 1168 upregulated genes and 929 downregulated genes in monocytes of HDM-allergic children with normal eosinophil counts (n=10) compared to healthy controls (n=14) (p-adj<0.05 and FDR<0.05). **(G)** Top overrepresented GO enrichment analysis and KEGG pathways showing upregulated (red) in classical monocytes from allergic-children with normal eosinophil counts (n=10) and healthy control (n=14). **(H)** Heatmap of proinflammatory related genes in HDM-allergic children and healthy controls in classical monocytes from HDM-allergic children (n=18) compared to healthy controls (n=14). **(I)** Monocytes from HDM-allergic children (n=17) and healthy controls (n=11) were treated with 10 ng/mL LPS for 4 hours prior to RNA sequencing analysis. Comparison of *TRAF2*, *IL17RA*, *TNFSF14*, and *TNFRSF25* expression profiles (log_2_[TPM+1]) in LPS-stimulated monocytes between HDM-allergic children and healthy controls.

We recruited 22 HDM-allergic children and 18 age-matched healthy controls, and their detailed general and clinical characteristics were recorded (**Table 1**). We measured the frequency of total monocytes and different subsets in the peripheral blood of HDM-allergic children and healthy controls. Human peripheral blood monocyte subsets are identified by flow cytometry through differential surface expression analysis of CD14 and CD16 (**Figure 1A**). The percentages and counts of total and classical monocytes (CD14^high^ CD16^-^) increased in allergic subjects. However, intermediate (CD14^high^ CD16^+^) and non-classical monocytes (CD14^low^ CD16^+^) showed no significant difference between the two groups (**Figures 1B, C**).

**Table 1:** General and clinical characteristics of healthy controls and HDM-allergic subjects.

Characteristics	Patients with Allergy (n=22)	Healthy controls (n=18)	P value
General characteristic			
Age (y), median (range)	4.42 (1.5-10.6)	6.78 (1.3-10.6)	0.616
sex (F/M)	13/9	9/9	0.565
Clinical data			
total IgE (KU/L), median (min-max)	546.41 (33-3043.22)	n.d.	
Der p sIgE (KU/L), median (min-max)	28.25 (0.35-100) *	n.d.	
Der f sIgE (KU/L), median (min-max)	27.27 (0.35-100) *	n.d.	
Allergic rhinitis (yes)	16	0	
Asthma (yes)	6	0	
Blood eosinophils, n (%)			
EOS>500	7/22		
EOS<500	15/22	18/18	

Upper limit value of specific IgE is 100KU/L.

n.d. no detection

The purified monocytes were subsequently subjected to RNA sequencing to investigate transcriptional regulation. Transcriptome comparison demonstrated that 979 genes were upregulated and 791 genes downregulated in allergic children’s monocytes (**Figure 1D**). KEGG pathway analysis showed that the upregulated genes in HDM-allergic children were enriched in the “regulation of cell adhesion”, “positive regulation of cytokine production”, and “regulation of I-kappaB kinase/NF-kappaB signaling” (**Figure 1E**). We also classified the HDM-allergic children into the high eosinophil group (>500/μL) and the normal eosinophil group by the absolute counts of eosinophils. The transcriptome comparison between allergic children with elevated eosinophil counts and healthy controls showed that 901 genes were upregulated and 640 genes were downregulated (**Supplementary Figure S1A**). The upregulated genes in children with elevated eosinophils were also enriched in the “regulation of small GTPase-mediated signal transduction” and “canonical NF−kappaB signal transduction” using KEGG pathway analysis (**Supplementary Figure S1B**). There were almost no differential genes between allergic children of the high eosinophil group and the normal eosinophil group (**Supplementary Figure S1C**). Surprisingly, the transcriptome analysis showed that 929 genes were downregulated and 1168 genes were upregulated even in allergic children with normal eosinophil counts compared to healthy controls (**Figure 1F**). KEGG pathway analysis also demonstrated that the upregulated genes were involved in the “small GTPase−mediated signal transduction”, “leukocyte cell−cell adhesion” and “canonical NF−kappaB signal transduction” (**Figure 1G**). Furthermore, the transcription levels of proinflammatory genes in HDM-allergic children with normal eosinophil counts were comparable to those in HDM-allergic children with elevated eosinophil counts. The expression of inflammatory-related genes such as *IL1A*, *IL6*, and *TNFAIP1* was upregulated both in allergic children of the high eosinophil group and the normal eosinophil group (**Figure 1H**). Monocytes derived from HDM-allergic children and healthy controls were subjected to low-dose LPS (10 ng/mL) stimulation for 4 h. Transcriptomic analysis revealed significant upregulation of proinflammatory mediators, including *TRAF2*, *IL17RA*, *TNFSF14*, and *TNFRSF25*, in the HDM-allergic cohort compared to healthy controls (padj<0.05; **Figure 1I**). The results indicate that monocytes in HDM-allergic children showed consistent transcriptional proinflammatory memory.

### H3K27me3 modulated the proinflammatory profile in monocytes of HDM-allergic children

**Figure 2:**
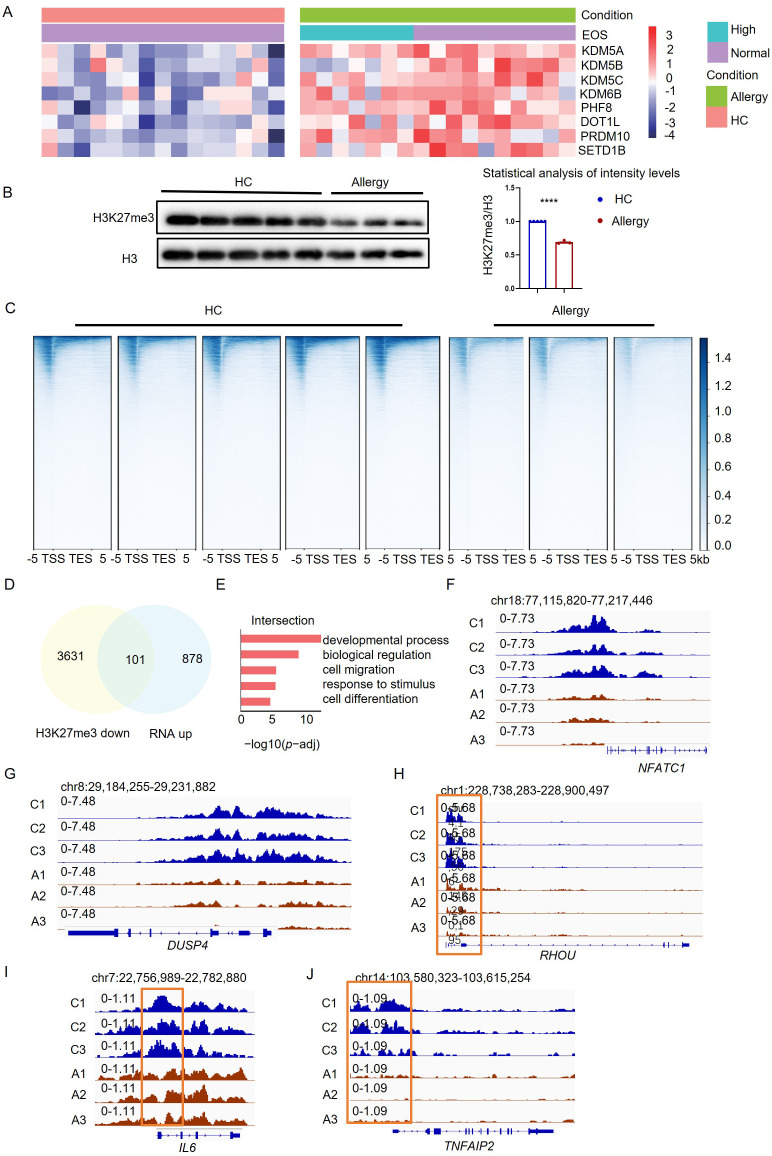
H3K27me3 methylation modulated the proinflammatory profile in monocytes of HDM-allergic children. **(A)** Heatmap visualization of eight differentially expressed histone-modifying enzyme genes in peripheral blood monocytes from HDM-allergic children (n=18) versus healthy controls (HC, n=14), demonstrating significant differential expression (false discovery rate-adjusted p-adj< 0.05). **(B)** Quantitative western blot analysis reveals decreased H3K27me3 methylation levels in monocytes of HDM-allergic children (n=3) compared to HC (n=5), with densitometric quantification of band intensities (ImageJ v1.53) (****p < 0.0001, unpaired two-tailed Student’s t-test). **(C)** Heatmap of H3K27me3 methylation across the whole gene body in monocytes of HDM-allergic children (n=3) and healthy controls (n=5) (p-adj<0.05), measured by CUT&Tag technology. **(D)** Intersection of transcriptional upregulated genes and downregulated genes of H3K27me3 methylation in monocytes of HDM-allergic children compared to healthy controls (p-adj<0.05). **(E)** GO enrichment analysis of the 101 intersection genes in **(D)** (p-adj<0.05). **(F-I)** CUT&Tag profiling of H3K27me3 chromatin occupancy at the *NFATC1*, *DUSP4*, *RHOU*, *IL6*, and *TNFAIP2* loci in peripheral monocytes from HDM-allergic children (n=3) versus healthy controls (n=5), with representative genome browser tracks (Integrative Genomics Viewer, v2.12.3) demonstrating differential histone modification patterns.

Epigenetic modification is the molecular basis of trained immunity (22). We applied RNA-Seq analysis to reveal the critical genes encoding histone-modifying enzymes, which were highly expressed in monocytes from HDM-allergic children. There were eight histone modification enzymes whose transcriptions were significantly changed in allergic children when compared to healthy controls (**Figure 2A**). Since the proinflammatory gene expression was elevated in trained monocytes, we focused on those enzymes that can activate gene transcription. Therefore, we chose KDM6B, which was upregulated in the monocytes purified from HDM-allergic children with or without elevated eosinophil counts, for further investigation (**Figure 2A**).

KDM6B is a demethylase of the H3K27me3 modification, which usually conducts gene suppression (23). We observed an attenuated H3K27me3 methylation in monocytes of HDM-allergic children, corresponding to the increased KDM6B expression (**Figure 2B**). We then applied CUT&Tag analyses of the H3K27me3 modifications on the chromatin of monocytes. Reduced H3K27me3 modification across the whole genome was detected in monocytes of allergic children compared to healthy controls (**Figure 2C**). This indicates that the transcriptional changes in monocytes in HDM-allergic children were probably due to the reduction of H3K27me3 level. To further validate the role of H3K27me3 modification, we then did integrated analyses by overlapping the transcriptionally upregulated genes based on RNA-seq results with genes covered by downregulated H3K27me3 peaks identified by CUT&Tag. There were 101 overlapped genes (**Figure 2D**). GO-enrichment of those overlapped genes showed biological pathways involved in “cell migration”, “response to stimulus” and “cell differentiation” (**Figure 2E**). H3K27me3 modification levels at the loci of immune-activating genes such as *NFATC1*, *DUSP4*, *RHOU* (**Figures 2F–H**) and proinflammatory genes such as *TNFAIP2* and *IL6* (**Figures 2I, J**) were significantly reduced in monocytes from allergic children compared to those cells from healthy controls. The results suggested that the reduced H3K27me3 modification in monocytes potentiates the persistent proinflammatory phenotype of HDM-allergic children.

### HDM-sensitized mice exhibited the same phenotype as HDM-allergic children

**Figure 3:**
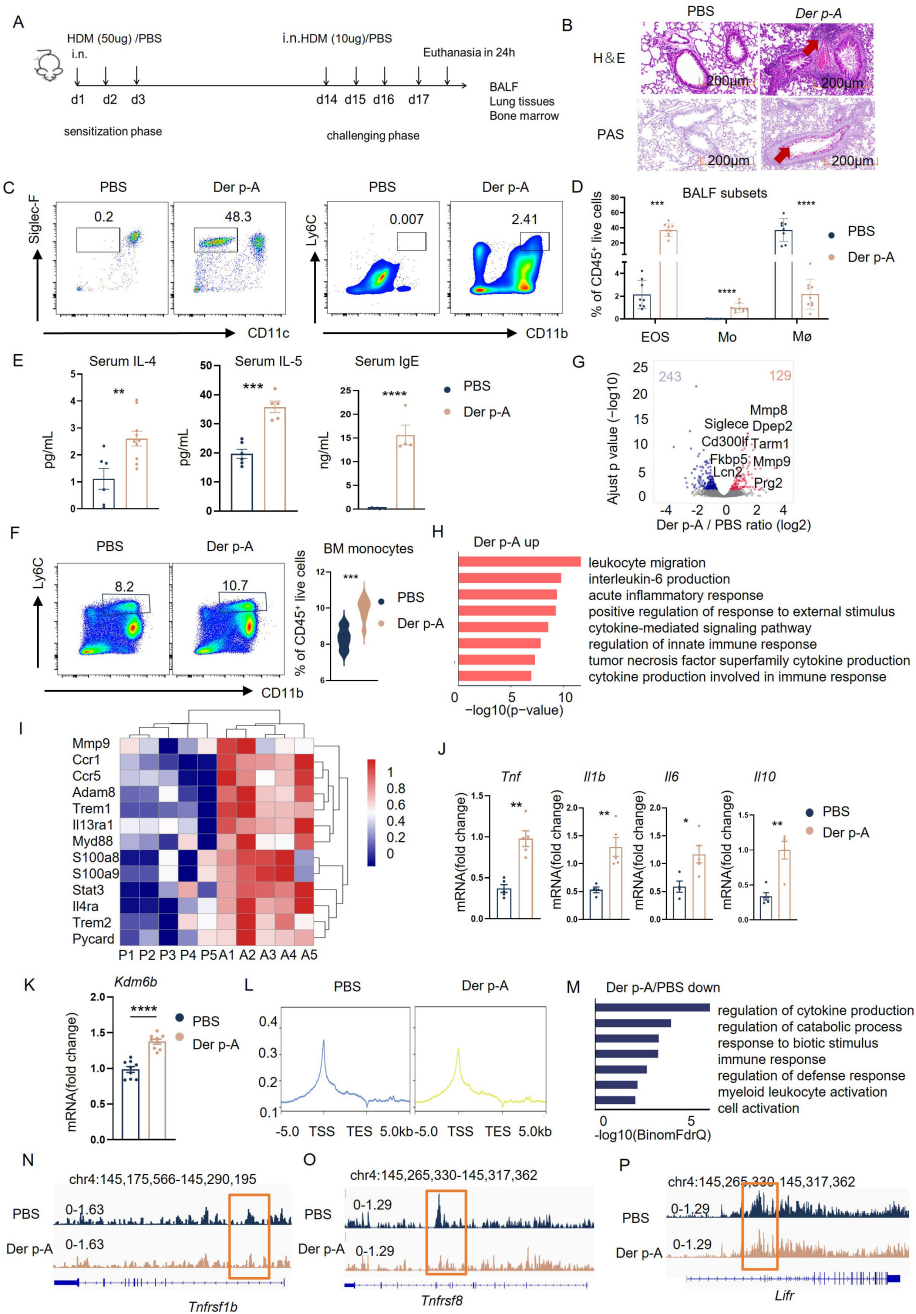
HDM-sensitized mice exhibited the same phenotype as HDM-allergic children. **(A)** Schematic of strategy to induce HDM-sensitized C57BL/6 mice. **(B)** Hematoxylin-eosin (H&E) and Periodic-acid-Schiff (PAS) staining of representative lung tissue in HDM-sensitized mice and PBS-controlled mice. Scale bar, 200 μm. **(C)** Gating strategies for eosinophils and monocytes in bronchoalveolar lavage fluid (BALF). Representative flow cytometry plots showing identification of eosinophils (CD45^+^CD11c^-^ Siglec-F^+^ live cells) and monocyte (CD45^+^CD11b^+^ Ly6C^+^ live cells) in BALF from PBS-controlled and HDM-sensitized mice (n=6-8). **(D)** Flow cytometric analysis of eosinophils (EOS), monocytes (Mo), and macrophages (Mø, CD45^+^CD11b^+^ F4/80^+^ live cells) in BALF from PBS-controlled and HDM-sensitized mice (n=6-8). **(E)** Statistical comparison of serum IgE, IL-4 and IL-5 secreation in PBS-controlled and HDM-sensitized mice (n=6-8). **p < 0.01, ***p < 0.001, ****p < 0.0001. p values were determined using unpaired two-tailed Student’s t-tests. **(F)** Comparison of monocytes (CD45^+^CD11b^+^Ly6C^+^ live cells) in bone marrow (BM) from PBS-controlled and HDM-sensitized mice (n=6-8) as determined by Flow cytometry. Data represent mean ± SEM of n>3 biological replicates. *p<0.05; **p<0.01 and *ns* no significance. p values were determined using unpaired two-tailed Student’s t-tests. **(G)** Volcano plots showing the 243 upregulated genes and 129 downregulated genes in bone marrow monocyte of HDM-sensitized mice compared to PBS-controlled mice (*p*-adj<0.05). **(H)** GO enrichment analysis of upregulated genes (red) in monocytes of HDM-sensitized mice (p-adj<0.05). **(I)** Heatmap of genes related to myeloid migration and immune activation in bone marrow monocytes from HDM-sensitized mice and PBS mice(p-adj<0.05). **(J)** Transcriptional comparison of *Tnf*, *Il6*, *Il1b* and *Il10* relative expression in bone marrow monocytes between HDM-sensitized mice and PBS-controlled mice, measured by Real-time fluorescence quantitative PCR. **(K)** statistical comparison of *Kdm6b* mRNA expression in bone marrow monocytes between HDM-sensitized mice and PBS-controlled mice, Data represent mean ± SEM of n>3 biological replicates. *p<0.05. **(L)** H3K27me3 signal across the gene body in monocytes of PBS mice and HDM-sensitized mice, measured by CUT&Tag technology. **(M)** GO enrichment analysis of downregulated genes in bone marrow monocytes from HDM-sensitized mice compared to PBS-controlled mice (*p*-adj<0.05). **(N-P)** CUT&Tag signal of H3K27me3 at the *Tnfrsf1b*, *Tnfrsf8*, and *Lifr* locus in bone marrow monocytes of HDM-sensitized mice and PBS mice.

To further confirm the epigenetic regulations in monocytes *in vivo*, we then established an acute HDM-sensitized mouse model to evaluate the critical role of KDM6B and related H3K27me3 modifications (**Figure 3A**). The histological examination of lung tissues from HDM-sensitized mice revealed distinct lung inflammation infiltration, as evidenced by H&E and PAS staining (**Figure 3B**). We observed increased monocytes (CD45^+^CD11b^+^Ly6C^+^) and eosinophils (CD45^+^CD11c^-^Siglec-F^+^) but decreased macrophages (CD45^+^CD11b^+^F4/80^+^) in lung tissues from HDM-allergic mice (**Figures 3C, D**). The secretion of IIL-4, IL-5 and IgE was significantly increased in serum of HDM-sensitized mice (**Figure 3E**). Increased monocytes were also found in the bone marrow of HDM-sensitized mice, indicating enhanced myelopoiesis (**Figure 3F**). We then purified the monocytes from HDM-sensitized mice for transcriptome analysis. Transcriptome comparison demonstrated that 129 genes were upregulated and 243 genes downregulated in HDM-sensitized mice (**Figure 3G**).

GO-enrichment analysis of RNA-seq results showed that the upregulated genes in monocytes of HDM-sensitized mice were involved in “leukocyte migration”, “interleukin-6 production” and “acute inflammatory response” (**Figure 3H**). Immune-activating genes such as *Ccr1*, *Ccr5*, *Trem1* and proinflammatory genes such as *Tnf*, *Il1b* and *Il6* were also upregulated in monocytes of HDM-sensitized mice compared to PBS mice (**Figures 3I, J**). The *Kdm6b* mRNA level was also upregulated in monocytes from HDM-treated mice compared to PBS-treated controls (**Figure 3K**).

Consequently, as we found in HDM-allergic children, we observed the downregulated H3K27me3 methylation in monocytes from allergic mice (**Figure 3L**). The downregulated H3K27me3 modifications enriched the genes involved in the “regulation of cytokine production,” “response to biotic stimulus,” and “immune response” using GO enrichment analysis (**Figure 3M**). H3K27me3 modification levels at the loci of proinflammatory genes such as *Tnfsf1b*, *Tnfrsf8* (**Figures 3N, O**) and cell activation genes such as *Lifr* (**Figure 3P**) were significantly reduced in monocytes from HDM-sensitized mice compared to PBS mice.

These results indicate that HDM-sensitized mice also exhibited the same transcriptional and epigenetic phenotype as HDM-allergic children.

### Selective inhibitor GSK-J4 attenuated the inflammatory response of monocytes stimulated with HDM

**Figure 4:**
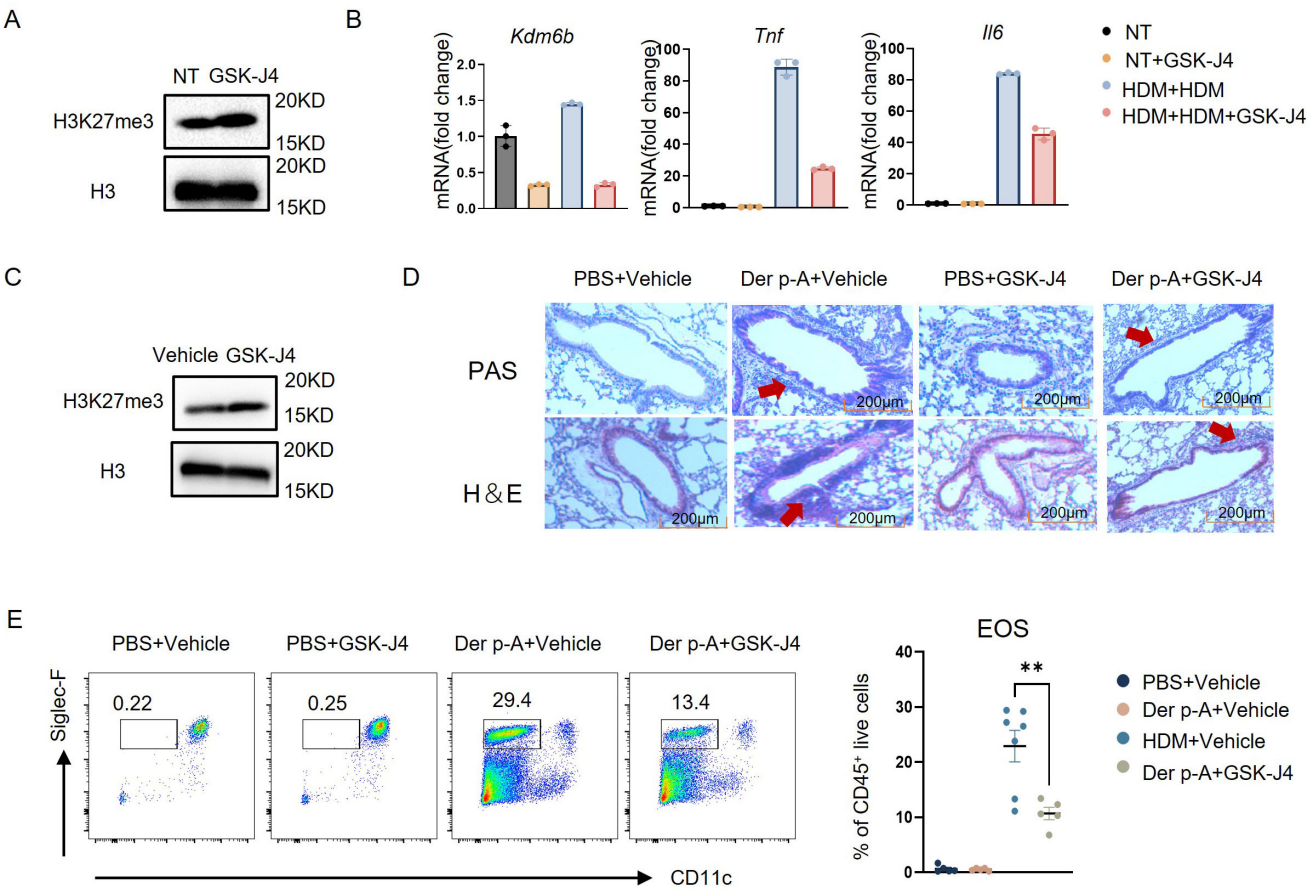
Selective inhibitor GSK-J4 attenuated the inflammation response of monocytes stimulated with HDM. **(A)** H3K27me3 expression levels in mouse bone marrow monocytes, treated with or without 5 μM GSK-J4, were analyzed by Western blot. **(B)** Experimental design for transcriptional regulation studies: Monocytes were divided into four treatment groups – (1) Untreated controls; (2) GSK-J4 monotherapy (24 h); (3) HDM priming (10 μg/mL, 16 h) followed by 24 h washout and re-stimulation (16 h); (4) HDM priming (16 h) with GSK-J4 co-treatment (5 μM, 24 h) prior to HDM re-challenge (16 h). Quantitative PCR analysis revealed significant alterations in *Kdm6b* (histone demethylase), *Tnf*, and *Il6* mRNA expression across treatment conditions. **(C)** H3K27me3 expression levels in total bone marrow cells from mice treated intraperitoneally with 20 mg/kg GSK-J4 or vehicle control for 14 days were analyzed by western blot. **(D)** Representative photomicrographs of lung tissue sections from the four experimental groups (PBS/Vehicle, PBS/GSK-J4, Der p-A/Vehicle, Der p-A/GSK-J4) stained with H&E and periodic PAS to assess inflammatory infiltration and mucus production, respectively. Scale bar: 200 μm. **(E)** Gating strategy for identifying eosinophils (CD45^+^CD11c^-^ Siglec-F^+^ live cells) in BALF from four groups by flow cytometry. Quantitative comparison of eosinophil frequencies across groups (n=5–7). Data are presented as mean ± SEM; **p < 0.01 (unpaired two-tailed Student’s t-test).

To illuminate the regulatory role of H3K27me3 in inflammatory gene expression, we employed a pharmacological inhibitor of KDM6B, GSK-J4, to treat bone marrow-derived murine monocytes. After GSK-J4 treatment, western blot confirmed the elevated H3K27me3 levels and concomitant reduction of *Kdm6b* mRNA (**Figures 4A, B**). Transcriptional profiling demonstrated that secondary HDM challenge significantly increased *Kdm6b*, *Tnf* and *Il6* expression compared to untreated controls, while GSK-J4 co-treatment attenuated the upregulation of these inflammatory mediators (**Figure 4B**).

To validate the therapeutic potential of KDM6B inhibition in house dust mite-induced allergic asthma, we conducted *in vivo* administration of GSK-J4 in a murine disease model. As evidenced by western blot, KDM6A/B-dependent H3K27me3 demethylation was successfully prevented in total bone marrow cells (**Figure 4C**). Compared to vehicle-treated counterparts, GSK-J4-treated mice had no significant alterations in general health status including body weight, and mature immune cell frequencies were maintained in normal range (**Supplementary Figure S3**). Subsequently, murine models of HDM-induced allergic asthma were employed. Histopathological analysis demonstrated significant attenuation of peribronchial inflammation and reduced mucus hypersecretion in GSK-J4-treated mice compared to vehicle-treated counterparts (**Figure 4D**). Quantitative flow cytometric analysis further showed a significant reduction in eosinophil frequency within lung tissues following KDM6B inhibition (**Figure 4E**). These results suggested that the selective inhibition of KDM6B attenuated the HDM-induced inflammation in monocytes.

## Discussion

Allergic diseases pose substantial clinical challenges due to the multifactorial nature of their pathogenesis and the difficulty in achieving complete healing. Although allergic diseases have been thought to be modulated by Th2 polarization and the crosslinking of IgE antibodies in basophils and mast cells for decades, emerging evidence highlights the critical role of innate immunity (24, 25). The increased monocyte proportion in persistently food-allergic children indicates the establishment of trained immunity. However, the mechanism and related pathological consequences have not been elucidated.

In this study, we aim to identify critical regulators for trained immunity in monocytes related to allergic diseases. In HDM-allergic children, we observed an increased monocyte population and proinflammatory transcriptional memory. Allergic diseases, such as asthma, are largely related to eosinophilic responses (26). Blood eosinophil count is also proposed as a predictive biomarker for disease severity and therapeutic outcomes (27, 28). Therefore, we classified the HDM-allergic children into a high eosinophil group and a normal eosinophil group based on blood eosinophil counts, and observed the same pro-inflammatory transcriptional phenotype in monocytes from both groups, regardless of elevated eosinophil counts (Figure). It further supports our hypothesis that, irrespective of disease status and severity, monocytes in HDM-allergic children consistently keep a memory state, sensitizing patients with recurrent disease onset.

Trained immunity is mediated by epigenetic regulation (10). We screened histone-modification enzyme genes using RNA-seq data from monocytes of HDM-allergic children, highlighting the H3K27me3 demethylase-KDM6B. KDM6B, also known as JMJD3, is an H3K27 demethylase that catalyzes the removal of the trimethylation mark (H3K27me3). H3K27me3 is a suppressive marker responsible for gene silencing (23). Thus, KDM6B facilitates transcriptional activation by modulating chromatin modifications, playing a critical role in the epigenetic regulation of development and differentiation (29, 30). The role of KDM6B in the trained immunity of monocytes remains poorly understood. In monocytes from HDM-allergic children, we observed decreased levels of H3K27me3, which inversely correlated with the upregulated transcription of pro-inflammatory and myeloid cell activation-related genes. These results align with the upregulation of activation and inflammatory genes at the transcriptional level found in monocytes from allergic patients, regardless of elevated eosinophil counts. Therefore, we propose that KDM6B modulates the establishment of trained immunity in monocytes from HDM-allergic children. To further test our hypothesis, we established an HDM-induced allergic mouse model. The HDM-sensitized mice displayed the same phenotype as HDM-allergic children. Increased monocytes and upregulated inflammatory gene expression were noted, along with a reduced H3K27me3 methylation level in monocytes from HDM-sensitized mice.

GSK-J4 is a selective dual inhibitor of the H3K27me2/me3-specific histone demethylases KDM6A (UTX) and KDM6B (JMJD3), which catalyze the removal of methyl groups from histone H3 lysine 27 residues (31). GSK-J4 stabilizes the repressive chromatin state and maintains the transcriptional silencing of proinflammatory genes in immune cells (32, 33). GSK-J4 treatment suppresses tumor growth and autoantibody production in SLE by reprogramming myeloid cells *in vivo (* 34–36). In this study, we applied GSK-J4 treatment both *in vivo* and *in vitro* to elucidate the regulatory role of KDM6B in monocytes from HDM-allergic animal models. We confirmed that the inhibition of KDM6B activity attenuated the activation of inflammatory genes regulated by trained immunity *in vitro*. *In vivo* administration of GSK-J4 also attenuated the inflammation response and eosinophil infiltration in the lung tissues of HDM-sensitized murine models. These findings align with prior studies demonstrating that inhibition of H3K27me3 demethylases ameliorates asthma pathology (37). The contribution of trained immunity to immunotherapy has been explored in recent studies, the inhibition or activation of histone modifying enzymes has been identified as another therapeutic target (22, 38, 39). The inhibition of KDM6B activity in monocytes may be the therapeutic target of HDM-allergic diseases. Although our data suggest KDM6B inhibition can attenuate allergic inflammation, other cell types besides monocytes may also participate in these effects, and it requires further validation via conditional knockout models.

As prenatal inflammation has been reported to increase asthma susceptibility in offspring (40), other studies have shown that infants who develop food allergies display a proinflammatory immune profile in cord blood (8). Increasing evidence indicates that trained immunity-mediated innate immune cell memory plays a vital role in allergic diseases. Our study demonstrated persistent inflammatory memory and KDM6B upregulation in recurrent HDM-allergic children. The KDM6B modulation may be responsible for the persistent inflammation, which is the driving regulator of recurrent onset of HDM-allergic symptoms. Selective inhibition of KDM6B activity and monocyte inflammatory response may be a therapeutic target in recurrent HDM-allergic diseases.

## Data Availability

The datasets presented in this study can be found in online repositories. The names of the repository/repositories and accession number(s) can be found in the article/**Supplementary Material**.
