# Supplementary material for: H3K27me3 modulates trained immunity of monocytes in HDM-allergic diseases
Source: Front Immunol. 2025 May 28;16:1572796. doi: 10.3389/fimmu.2025.1572796 (PMC12152424; doi:10.3389/fimmu.2025.1572796)
Supplement: Supplementary file 1 [file Table1.docx]

*Supplementary material*

H3K27me3 modulates trained immunity of monocytes in HDM-allergic diseases

Lingli Han *et al*

Corresponding author’s email: [jinqiaosun@fudan.edu.cn](mailto:jinqiaosun@fudan.edu.cn)


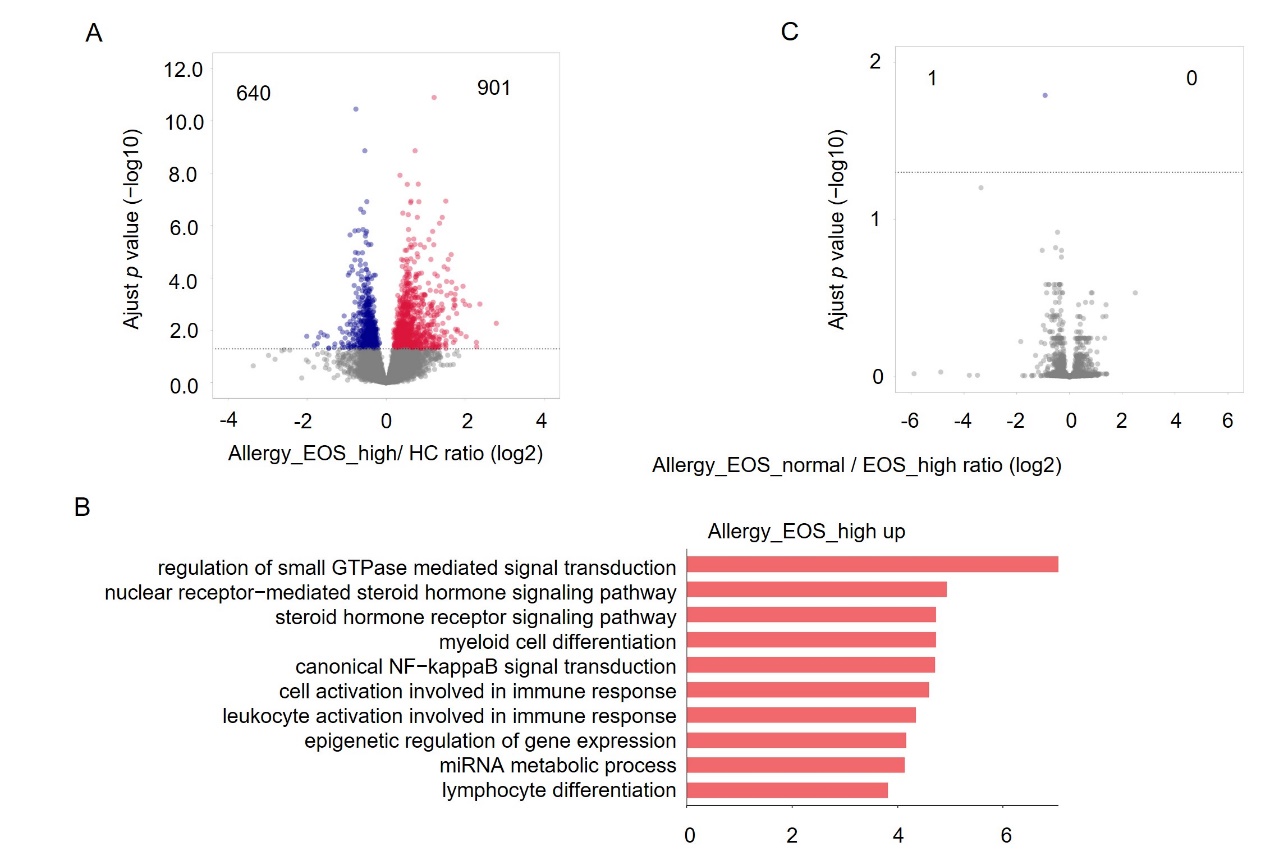


Figure S1 Monocytes showed persistent transcriptional pro-inflammatory memory in HDM-allergic children.

(A) Volcano plots showing the 901 upregulated genes and 640 downregulated genes in monocytes of HDM-allergic children with elevated eosinophil counts (n=8) compared to healthy controls (n=14) (p-adj<0.05). (B) Top overrepresented GO enrichment analysis and KEGG pathways showing upregulated (red) in classical monocytes from allergic-children with high eosinophil counts and healthy control. (C) Volcano plots showing no upregulated genes and 1 downregulated gene in monocytes of HDM-allergic children with elevated eosinophil counts (n=8) compared to HDM-allergic children with normal eosinophil counts (n=10) (p-adj<0.05).


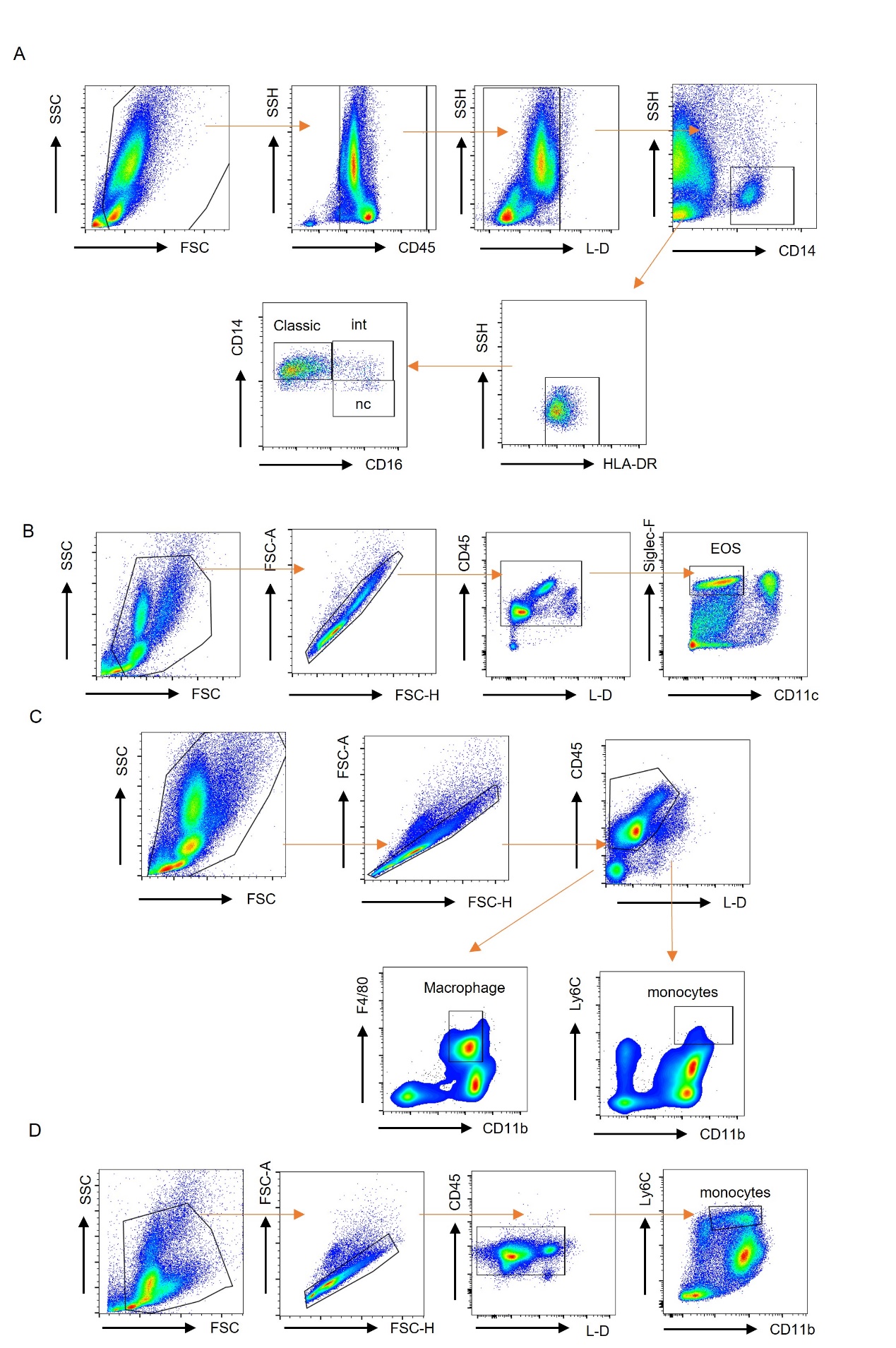


Figure S2 Flowcytometric gating strategy in human subjects and mouse models.

(A) Representative fluorescence-activated cell sorting (FACS) plots for the identification of classical (CD14^high^CD16^-^), intermediate (CD14^high^CD16^+^) and non-classical monocytes (CD14l^ow^CD16^+^) in HDM-allergic children and healthy controls. (B) Representative FACS plots for the identification of eosinophils (CD45^+^CD11c^-^Siglec-F^+^live cells) in BALF from HDM-sensitized mice and PBS-controlled mice. (C) Representative FACS plots for the identification of macrophages (CD45^+^CD11b^+^F4/80^+^ live cells) and monocytes (CD45^+^CD11b^+^Ly6C^+^ live cells) in BALF from HDM-sensitized mice and PBS-controlled mice. (D) Representative FACS plots for the identification of monocytes (CD45^+^CD11b^+^Ly6C^+^ live cells) in Bone marrow from HDM-sensitized mice and PBS-controlled mice.


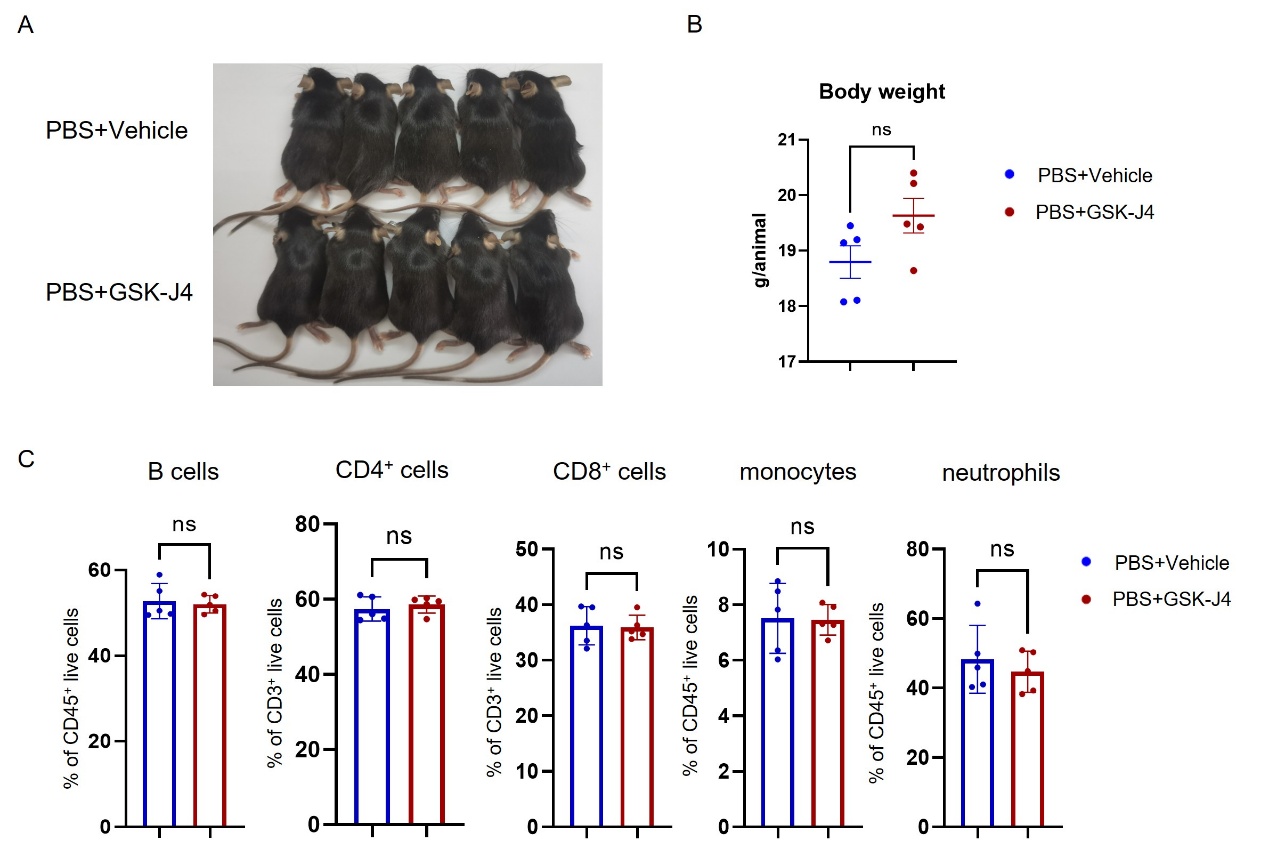


Figure S3 GSK-J4 administration does not alter systemic health or mature immune cell homeostasis in *vivo*

(A) Representative photographs depicting the gross morphology of GSK-J4-treated and vehicle-treated mice. (B) Body weight monitoring. Analysis of body weight in GSK-J4-treated (20 mg/kg, 14 days) versus vehicle-treated mice (n=5). Data are presented as mean ± SEM; *ns* no significance (unpaired two-tailed Student’s t-test). (C) Flow cytometric analysis revealed no significant differences in the frequencies of splenic lymphocytes—including B cells (CD45⁺B220⁺), CD4⁺ T cells (CD45⁺CD3⁺CD4⁺), and CD8⁺ T cells (CD45⁺CD3⁺CD8⁺)—or bone marrow myeloid subsets (monocytes: CD45⁺CD11b⁺Ly6G⁻; neutrophils: CD45⁺CD11b⁺Ly6G⁺) between mice treated with 20 mg/kg GSK-J4 and vehicle-treated controls (n=5). Data are presented as mean ± SEM; ns, not significant (unpaired two-tailed Student’s t-test).
